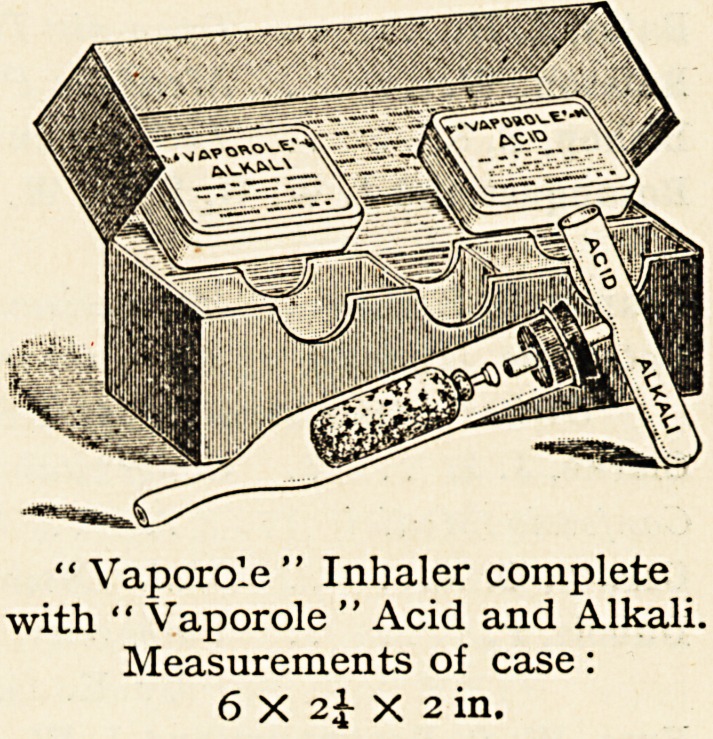# Notes on Preparations for the Sick

**Published:** 1910-03

**Authors:** 


### Notes on Preparations for the Sick.

**"Tabloid" Thyroid Gland** (Standardised).—BURROUGHS, WELLCOME & Co., London.—Represents the whole substance of carefully selected, healthy glands of the sheep, and contains the unaltered, undiminished, essential activity of the normal thyroid gland.

This tabloid is so standardised by chemical means controlled by physiological test as to ensure that the desiccated gland substance, of which each represents a definite amount, contains not less than 0.2 per cent. of iodine in organic combination.

The following strengths are available :—

gr. ½. gr. 1½. gr. 2½. gr. 5.

**"Tabloid" Pituitary Gland,** gr. ii.—BURROUGHS, WELLCOME & Co., London.—Extracts of the infundibular part of the gland produce a prolonged rise of blood-pressure, a slowing and strengthening of the heart beat, an increased secretion of urine, and contraction of the uterus. In acromegaly the pituitary gland is generally found diseased. The gland substance is employed in acromegaly, and has been reported to relieve the headache and the neuralgic pains in the limbs, whilst in other cases memory has improved and lethargy diminished. The benefit appears to be increased in some cases by the combined use of pituitary gland with thyroid gland.

**Thymoform Tablets.**—OPPENHEIMER, SON & Co., London.—These tablets form a pleasant, non-irritating method of exhibiting the powerful antiseptic, deodorant and bactericidal properties of Thymol and Formaldehyde.

They are prepared in the form of tablets, with a pleasantly flavoured basis of cane sugar, and are quite agreeable to taste. When sucked slowly they dissolve in the saliva, and thus form an effective disinfectant, which is applied to the whole oral cavity and throat, and is capable of exerting a continuous bactericidal action, which is much more effective and lasting than gargles or mouth washes. In septic conditions of the mouth, whether arising from defective teeth or disease generally, they are ideal, removing the fœtor rapidly, and leaving a pleasant, clean taste in the mouth which is appreciated by the patient.

---

**Salen.**—Society of Chemical Industry in Basle, 6 Harp Lane, London. E.C.—Salen, a mixture of the methyl and ethyl glycolic acid esters of salicylic acid, is an oily liquid readily soluble in alcohol, ether and castor oil, sparingly soluble in olive oil, more readily in a mixture of olive oil and castor oil, or of chloroform and olive oil in equal parts.

Salen is odourless, *perfectly non-irritating*, stable, and inexpensive.

Its freedom from irritating properties makes it possible to rub Salen into the skin by careful massage, to leave it in contact with the skin for some time, and to apply it continuously to the painful part.

The following modes of application are advised :—

℞ Salen. pur. 15 gms. "To be applied with a brush" (in gout).

| | |
|---|---|
| ℞ Salen.<br>Spirit rec. āā 10 gms.<br>℞ Salen. 10 gms.<br>Chloroform.<br>Ol. Olivæ āā 5 gms. | "The painful parts to be rubbed two or three times daily with $\frac{1}{2}$ to 1 teaspoonful, and then to be covered with cotton wool or flannel." |

---

**Aperitol.**—The J. D. Riedel Co., 54 Cannon Street, London.—The drug so named is a mild, non-griping and harmless laxative. Chemically it is described as a valerianate of phenolphtalein.

Aperitol is a white powder, absolutely tasteless, and insoluble in water and dilute acids, but is gradually resolved into its components by weak alkalies. Hence it passes through the stomach unaltered, whilst in the intestines valerianic acid and phenolphtalein are liberated, the latter of which is almost wholly excreted in the fæces.

The ordinary dose is six grains, two tablets or bonbons of three grains each. This dose commonly causes only a single action of the bowels, and it does not appear to lose its efficacy after repeated administration. Its action, owing to the presence of the sedative valerian, is entirely free from pain.

**"Vaporole" Ammonium Chloride Inhaler.**—BURROUGHS, WELLCOME & Co., London.—The "Vaporole" Ammonium Inhaler consists of a compact apparatus which will, whenever required, deliver perfectly neutral vapour of pure and freshly-prepared Ammonium Chloride.

Showing "Vaporole" Inhaler in use.

Many advantages are possessed by the "Vaporole" Inhaler over the ordinary forms. It has no rubber connecting-tubes to deteriorate, loosen, or become clogged with crystals of Ammonium Chloride. Supplies of acid and alkali sufficient for each application are carried separately in hermetically-sealed "Vaporole" containers, and will maintain their full strength and remain instantly available for any length of time until required for use. Thus with the "Vaporole" Inhaler there are no cumbersome bottles, no stock liquids of a dangerous and volatile nature, and no glass stoppers to get detached, misplaced or lost. The compactness, simplicity and efficiency which characterise the whole apparatus ensure portability, facilitate application, and make for certainty of result.

Each "Vaporole" Inhaler, together with a supply of "Vaporole" Acid and "Vaporole" Alkali, is securely packed in a neat case.

"Vaporole" Inhaler complete with "Vaporole" Acid and Alkali. Measurements of case: $6 \times 2\frac{1}{4} \times 2$ in.

## Figures and Tables

**Figure f1:**
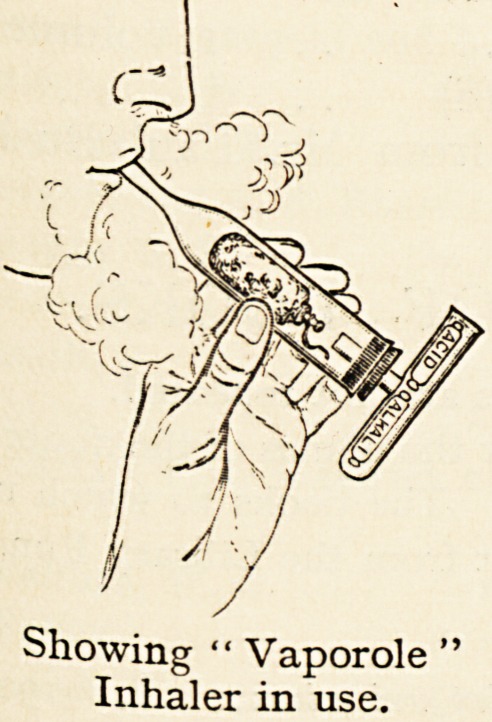


**Figure f2:**